# Squatter Sovereignty

**Published:** 1859-08

**Authors:** 


### SQUATTER SOVEREIGNTY.

Right much tickled was our national pride, when once upon a time, some live Yankee, concocted a fib, in order to pass the customs, or for some other reason, by telling the wondering listener that in America, all were sovereigns.

There is a party of ragamuffins in our land, who are always contending for the "largest liberty;" the liberty of free love for example; the liberty of short measure, scant weight, and the like; the liberty of cheating your neighbor "in the way of trade;" among others, there are not a few contending for the liberty of locating their lazy bones on any unclaimed vacant land of the general government, asserting the right thereby, of becoming the lawful owners of the same. So there are a good many squatters and a good many sovereigns in these most free, and most enlightened "states" of North America. There is, however, another class increasingly large, which are both squatters and sovereigns; but a peculiarity about them is, that they assert the right, and practice the same, only on Sundays; most numerous in high places. Go to any Congregational or Presbyterian church in or about Union Square, Fifth Avenue, or Fourteenth street on any fine Sunday; take position in the gallery; and for the time, the hour of prayer; see well dressed, stalwart, lazy men, firmly seated, forehead resting on the pew back in front, an arm for a pillow; with elbows to match, stretched along for a yard! How much of the prayer does any of these gentry hear of a hot summer's day, in a position so sleep inviting? Or if "wide awake," in devising some stock jobbing plan for next day, what a cosy attitude for profound thought, for laying pipes and traps to perfection! Out upon it, ye lazy brood! pity it is that there is not another with a good bundle of cords, to whip you out of the "Father's House."

The Episcopal service, more decent and more wise, exacts a frequent change of position, thus preventing that stagnation of blood which induces sleep. Our women, with their present constitutions and habits, are physically incapacitated in most cases for standing still beyond a very few minutes; but they are of a more wakeful, of a more devotional nature, and have greater self control as to the proprieties of life; hence to them, sitting in time of prayer, is more allowable; but that any one pretending to be a man, should spraddle his great hulk and bottom "just so," is a disgrace and an abomination.

